# Towards a new therapeutic approach based on selection for function in tumors: response to Dr. Mesut Tez

**DOI:** 10.1093/emph/eoae029

**Published:** 2024-11-28

**Authors:** Frédéric Thomas, James DeGregori, Andriy Marusyk, Antoine M Dujon, Beata Ujvari, Jean-Pascal Capp, Robert Gatenby, Aurora M Nedelcu

**Affiliations:** CREEC/CANECEV, MIVEGEC (CREES), University of Montpellier, CNRS, IRD, Montpellier, France; Department of Biochemistry and Molecular Genetics, University of Colorado Anschutz Medical Campus, Aurora, CO, USA; Department of Tumor Microenvironment and Metastasis, H Lee Moffitt Cancer Center and Research Institute, Tampa, FL, USA; CREEC/CANECEV, MIVEGEC (CREES), University of Montpellier, CNRS, IRD, Montpellier, France; School of Life and Environmental Sciences, Deakin University, Waurn Ponds, Geelong, VIC 3216, Australia; School of Life and Environmental Sciences, Deakin University, Waurn Ponds, Geelong, VIC 3216, Australia; Toulouse Biotechnology Institute, University of Toulouse, INSA, CNRS, INRAE, Toulouse, France; Department of Tumor Microenvironment and Metastasis, H Lee Moffitt Cancer Center and Research Institute, Tampa, FL, USA; Department of Biology, University of New Brunswick, Fredericton, New Brunswick, Canada

We would like to express our gratitude to Dr. Mesut Tez for his thoughtful and positive response to our article ‘A new perspective on tumor progression: Evolution via selection for function’, recently published in *Evolution, Medicine, and Public Health* [1]. Dr. Tez’s reflections not only align with our main arguments but also enrich the discussion by drawing parallels between our work and Sir Richard Peto’s black box epidemiology, as well as Stuart Kauffman’s autocatalytic models [2, 3].

We are particularly pleased that Dr. Tez recognizes the centrality of selection for function at the tumor level, in addition to the traditional Darwinian view of tumor progression based purely on the fitness of individual cells. His framing of cancer cell populations as ‘quick problem-solvers’ adept at navigating hostile environments resonates strongly with the collective adaptability and functional redundancy we sought to highlight through the ecological concept of group phenotypic compositions (GPCs) [4], that we previously adapted to tumors [5].

The emphasis Dr. Tez places on the therapeutic implications of disrupting key functional traits and networks within tumor ecosystems is of great significance. As he points out, this framework opens the door to novel treatment strategies aimed at targeting the adaptive mechanisms that bolster a tumor’s capacity for resilience. By focusing on the cooperative networks and redundant pathways that enable tumors to thrive, we believe that oncology could make significant strides in improving patient outcomes.

In light of Dr. Tez’s insightful contribution, we feel further compelled to explore how these cooperative dynamics within tumor populations might be destabilized by carefully designed and timed interventions. The idea of disrupting oncogenic GPCs to reduce a tumor’s capacity for rapid problem-solving indeed represents a paradigm shift in cancer treatment strategies. It is an exciting avenue for future research, and we wholeheartedly agree that a deeper understanding of these non-linear, self-organizing systems could provide new opportunities for therapeutic breakthroughs.

We greatly appreciate Dr. Tez’s engagement with our work and the stimulating ideas he has brought forward. His response not only validates our proposed shift towards a function-based evolutionary perspective on cancer progression, but also opens new paths for exploration, particularly regarding therapeutic applications.

Thank you once again for the opportunity to respond to this excellent commentary, and we look forward to the continued discussion on this important topic.
